# FTO associations with obesity and telomere length

**DOI:** 10.1186/s12929-017-0372-6

**Published:** 2017-09-01

**Authors:** Yuling Zhou, Brett D. Hambly, Craig S. McLachlan

**Affiliations:** 1 Rural Clinical School, University of New South Wales, Sydney, 2052 Australia; 2 Discipline of Pathology and Bosch Institute, University of Sydney, Sydney, Australia

**Keywords:** Obesity, Genetic polymorphism, Nutrient sensing, FTO SNPs, Telomere length, Energy balance

## Abstract

This review examines the biology of the Fat mass- and obesity-associated gene (FTO), and the implications of genetic association of FTO SNPs with obesity and genetic aging. Notably, we focus on the role of FTO in the regulation of methylation status as possible regulators of weight gain and genetic aging. We present a theoretical review of the FTO gene with a particular emphasis on associations with UCP2, AMPK, RBL2, IRX3, CUX1, mTORC1 and hormones involved in hunger regulation. These associations are important for dietary behavior regulation and cellular nutrient sensing via amino acids. We suggest that these pathways may also influence telomere regulation. Telomere length (TL) attrition may be influenced by obesity-related inflammation and oxidative stress, and FTO gene-involved pathways. There is additional emerging evidence to suggest that telomere length and obesity are bi-directionally associated. However, the role of obesity risk-related genotypes and associations with TL are not well understood. The FTO gene may influence pathways implicated in regulation of TL, which could help to explain some of the non-consistent relationship between weight phenotype and telomere length that is observed in population studies investigating obesity.

## Background

Genetic background is important in understanding metabolic pathways that regulate adiposity [1, 2]. Telomere length attrition may be influenced by obesity-related inflammation and oxidative stress. There is emerging evidence to suggest that telomere length (TL) and obesity are bi-directionally associated [3–5]. However, the role of regulatory obesity genotypes and associations with TL are not well understood. Across genetic association studies of obesity, there is considerable obesity phenotype variance [6, 7]. Factors such as gene drift, environment, migration or ethnicity may in part be responsible for such obesity phenotype variance [8–10]. Alternatively, TL-obesity relationship could theoretically be influenced by gene methylation levels, which in turn could affect genes, due to the characteristics of, for example, permitting changes which affect gene function but at the same time do not modify DNA sequences. The fat mass and obesity associated (FTO) gene is a likely example of a regulatory “master switch” gene that influences epigenetic control over a number of key regulatory pathways in obesity regulation. We have recently demonstrated that FTO genotype is not associated with FTO methylation levels [11]. In order to understand how FTO may be associated with TL it is important to consider the role of the FTO gene in the context of its function, regulation and obesity.

FTO has been correlated with metabolic syndrome and diabetes risk [12, 13]. The genetic link to metabolic balance and adiposity homeostasis is important for chronic diseases and also regulation of telomere length [9, 13, 14], since there is an association between shorter telomeres and increased body mass index, increased adiposity, and increasing waist to hip ratio and visceral excess fat accumulation [15]. Additionally, the biochemical abnormalities of obesity, for example, abnormal glycemic and lipidemic profiles, lead to organ dysfunction resembling the accelerated aging process [15]. An emerging hypothesis links obesity, shorter telomeres and accelerated aging [16], however, the manner in which the FTO gene fits into this hypothesis remains to be fully elucidated.

## GWAS and FTO associations with obesity

The FTO gene expresses a 505 amino acid protein that shares sequence motifs with Fe(II)- and 2-oxoglutarate (2-OG) dependent dioxygenases [17, 18]. This enzyme family can repair alkylated DNA and RNA by oxidative demethylation, an important mechanistic process in epigenetic regulation of genes [18–22], suggesting that FTO may be involved in DNA/RNA demethylation [21].

FTO has been clearly identified as an obesity associated gene via Genome Wide Association Study (GWAS) [23–25]. The application of GWAS led to the understanding that a number of variants in the first intron of the FTO gene may be associated with adiposity, for example, each additional copy of the rs9939609 risk allele is associated with increased BMI of ~0.4 kg/m^2^ [23–25]. Subsequently, at least 75 obesity susceptibility loci [9, 26] have been described across the genome. However, FTO appears to have a greater effect on obesity compared to all other obesity loci and this has been confirmed through replication studies throughout the life span and across ethnicities [27–32].

The FTO rs9939609 SNP is the most commonly reported population obesity gene in association studies. The risk allele (A allele) of rs9939609 is associated with greater total energy (food intake), and increased protein and fat intake in children and adults [33–36]. FTO SNPs, mainly located in intron 1, have been reported to be associated with individual variation in appetite rating scales, loss of control over eating, as well as eating in the absence of hunger [33, 35, 37–47]. It has been observed that children and adolescents that carry the FTO rs9939609 A allele (AA or AT genotype) self-select more energy dense components of a test meal [45, 46]. In adults, the A allele carriers are more likely to demonstrate a loss of control of eating [46], altered postprandial satiety levels [39], increased feeling of hunger [47] and reduced fullness self-ratings [48]. The FTO rs9939609 AA genotype is associated with activity of neural substrates associated with food-cue reactivity [49–51].

## FTO associations with amino acids

Amino acids play a role in central regulation of food intake - the understanding how amino acids may influence FTO signaling is not well understood. FTO catalyzes the oxidation reaction of methyl DNA/RNA substrates, together with co-factors 2-OG, Fe (II) and oxygen [52]. Early studies presumed that FTO likely functions as an intracellular sensor by assessing the concentration of 2-OG, which is a key intermediate of the citric-acid cycle. Studies using mouse and human cell lines demonstrated that by restricting total amino acids in the medium there was significant downregulation of mRNA and protein expression of FTO in vitro [53], consistent with FTO being a sensor of amino acids, instead of possibly being a 2-OG, within cells [54].

The importance of amino acids are starting to be recognized in metabolic systems, for example, a reduction in leucine in mouse models increases hepatic insulin sensitivity via general control non-depressible 2 (GCN2) /mammalian target of rapamycin (mTOR) and AMP-activated protein kinase (AMPK) pathways [55]. These pathways involve nutrient-responsive protein kinases and are important for proper regulation of glucose metabolism in mammals at both the hormonal and cellular level (see below) [56]. Phenylalanine is also a significant circulating amino acid that declines with weight loss [57], although there is a lack of evidence to suggest a direct interaction with FTO pathways. For example, fasting over 72 h results in an increase in phenylalanine and an associated decrease in skeletal mTOR activity and cell growth signaling [58].

## FTO signaling pathways

### FTO regulates energy sensors in the central nervous system

Global energy sensors in the central nervous system include mammalian target of rapamycin (mTOR), AMP-activated protein kinase (AMPK), and uncoupling protein 2 (UCP2) [59]. Interestingly, these three sensors are all likely to interact with the FTO gene both directly or indirectly. FTO-overexpressed cells are insensitive to amino acid deficiency under the regulation of the mammalian target of rapamycin complex 1 (mTORC1) signaling pathway, a major regulator of cell growth and basic catabolic mechanisms [60, 61]. As eluded to above, while there is an association between mTOR and phenylalanine in fasting states, FTO obesity-risk SNPs are reported to affect AKT expression of interacting protein (AKTIP) in an allele-dosage manner by altering the binding site of the transcription factor Cut Like Homeobox 1 (CUX1) [62]. This influences the phosphorylation of AKT’ regulation sites, likely modulating AKT activity via an AKT-AKTIP direct interaction [63].

### FTO-downstream mTORC1 influences obesity

The mTORC1 complex itself plays a complex role in obesity. For example, DEP-domain containing mTOR-interacting protein (DEPTOR) functions to suppress mTORC1’s function [64]. Consequently, this inhibits the mTORC1 negative feedback loop, resulting in increased insulin signaling and Akt/PKB activation [64]. Mechanisms underlying FTO expression regulation by amino acids and interactions with mTORC1 are not entirely clear. A primary hypothesis is that FTO is an amino acid (AA) sensor coupling AA levels to mTORC1 [65]. mTORC1 is involved in cell cycle regulation, for example, nutritional signaling through target of rapamycin complex 1 (TORC1) in yeasts permits a cellular check point regulatory choice for a cell to either continue replication in the presence of DNA damage or cell cycle arrest to maintain genetic stability [66]. Furthermore, because of the role of mTORC1 in genetic stability, it is possible that mTORC1 will also have an influence on telomere regulation [67]. FTO SNPs have been clearly associated with greater food intake and increased hunger, but not with decreased resting energy expenditure or low physical activity in human population studies [68]. This paradox of FTO function might be explained by the interaction of FTO with diverse functions of mTORC1.

The metabolism of glucose and glutamine, and primary carbon sources utilized by mitochondria, is directly regulated by mTORC1 [69]. The activity of the mTOR kinase itself, an essential component of mTORC1, is increased by cellular adenosine triphosphate (ATP) levels [70]. Additionally, FTO-variant linked obesity may be associated with altered metabolic functions through activation of downstream metabolic mediators including AMPK [71]. The gastrointestinal tract “hunger hormone”, ghrelin, alters hypothalamic mitochondrial respiration in neurons in an UCP2-dependent manner. This process is driven by a hypothalamic fatty acid oxidation pathway, that involves AMPK, CPT1 and free radical scavenging by UCP2 [72]. Furthermore, mTORC1 activation is associated with increased oxygen consumption [73]. In summary, we hypothesize that global energy sensors, AMPK, UCP2 and mTORC1, are likely to be influenced by FTO gene regulation, supporting the hypothesis that FTO functions in the process of obesity.

### IRX 3 and IRX 5 bridge FTO and obesity

An alternate explanation for the discrepancy in FTO SNP polymorphisms and regulation of energy expenditure is via the effect of the obesity-associated FTO region on expression regulation of homeobox gene IRX3 (Iroquois homeobox protein 3) and IRX5. A SNP within intron 1 of the FTO gene alters the expression of IRX3 and IRX5 to regulate adipocyte thermogenesis via influencing adipocyte differentiation [74]. Indeed, Irx3-deficient mice were found to lose 25 to 30% of their weight, due to an increase in basal metabolic rate. The hypothalamic expression of a dominant-negative form of Irx3 reproduces the metabolic phenotypes of Irx3-deficient mice [75], suggesting hypothalamic expression of FTO possibly plays an important role in adiposity regulation via Irx3 pathways.

### FTO and hunger-related hormone signaling

Interestingly, mTORC1 is a major intracellular target for hormones and nutrients that regulate food intake and body weight in the hypothalamus [73]. Additionally, hypothalamic CUX1 expression influences leptin receptor trafficking, resulting in altered leptin signaling in mice, which modulates eating behavior [76]. These findings provide more evidence to explain the diverse influence of FTO SNPs on energy balance regulation.

Both mouse Fto and human FTO mRNA are expressed ubiquitously, but FTO expression is higher in the brain and specifically the hypothalamus [18, 77]. This regional distribution for expression is intriguing because the hypothalamus plays a key role in the regulation of both energy balance and control of food intake.

There is a lack of support for the idea that human FTO expression is regulated at the transcriptional level in a leptin-dependent manner. Leptin inhibits hunger, and if it has an effect it appears to be indirect. The relationship between leptin and FTO expression has been explored extensively in rodents, but no clear consensus emerges concerning this relationship. For example, leptin reduces FTO expression in the hypothalamus by activating the STAT3 signaling pathway, in which the Leptin Receptor Long Isoform (LepRb) is also required [78]. Paradoxically, Fto knock-out mice develop features of metabolic syndrome that are normally observed in leptin deficient mice [37]. Mice studies have identified that a fed state is associated with increased hypothalamic Fto mRNA expression, while extended caloric restriction reduces hypothalamic Fto protein expression in Leptin-knockout mice (Lepob) [18, 79]. This latter response is absent in Leptin receptor-mutated mice (Leprdb) [79]. In contrast, Fto expression is increased within the hypothalamus of food-restricted and food-deprived rats [80]. Interestingly, an alternate observation reveals an over-expression of Fto mRNA and protein in the rat hypothalamus due to 48 h fasting [81], whilst 40% reduced Fto expression in rat arcuate nucleus of the hypothalamus increases food intake by 16% [82]. These cumulative findings may be explained by a difference in mRNA versus protein FTO expression, as a consequence of complex FTO gene regulatory mechanisms.

In the transgenic Fto-overexpressing mouse model, greater food intake induces a gene-dose-response increase in body fat mass [83]. Weight gain in humans may be because of an endocrine balance shift from the satiety hormone leptin toward the hunger-promoting hormone ghrelin. Intriguingly, Karra et al. observed rs9939609 AA and TT genotypes showed divergent neural activity in response to circulating ghrelin, which is a key mediator of ingestive behavior [49]. In summary, these data support the concept of fine tuning of hypothalamic neurons to facilitate metabolic regulation by altering their own activities or the activities of upstream/downstream targets in response to hormonal and nutrient signals.

## FTO and TL regulation

### Fe(II)- and 2-OG dependent dioxygenase family and TL regulation

Fe(II)- and 2-OG dependent dioxygenase family members are involved in diverse processes, including DNA and RNA repair, fatty acid metabolism, posttranslational modifications and demethylation of CpG islands [84, 85]. CpG methylation is associated with gene silencing or CpG demethylation with increased transcription. It is possible that a methylation or demethylation status switch that alters expression of TL regulation-related genes, via 2-OG dependent dioxygenase, is involved in telomere attrition process. The relationship between FTO, a 2-OG dependent dioxygenase, and telomere length regulation is still not elucidated.

One known 2-OG dioxygenase family of proteins that has been associated with regulating TL is the Ten-Eleven-Translocation (TET) proteins, Tet1, Tet2 and Tet3 [86]. Tet proteins act as DNA demethylation enhancers that can influence telomere homeostasis. Functionally, mouse embryonic stem cells deficient for all three Tet proteins (Tet triple knockout) have been shown to exhibit increased telomere-sister chromatid exchange and elongated telomeres [86]. Similar to Tet, the 2-OG dependent dioxygenase catalytic activity of FTO may regulate gene transcription or TL regulation via nucleic acid demethylation. For example, DNA methylation can directly affect transcription factor binding, or indirectly change post-translational histone packaging and modulation of chromatin conformation and function [87, 88]. Identification of epigenetic modifications may aid the exploration of genotype-phenotype interactions in metabolic disease relevant to obesity and telomere regulation. Gerken et al. has made the valid observation that breakdown of genomic repair processes may be associated with susceptibility to obesity and metabolic syndrome [18].

### FTO regulates TL indirectly

Interestingly, FTO may influence TL regulation via expression of upstream/downstream flanking genes. For example, FTO rs8050136 (without altering FTO expression) correlates with the expression of retinoblastoma-like 2 protein (Rbl2) gene [89]. The Rbl2 gene is approximately 270,000 base pairs distant from FTO [89]. Rbl2 inhibits Dnmt3a,3b expression, and interestingly this interaction influences telomere regulation, involving significant genomic hypomethylation, including in the subtelomeric regions. Telomeric phenotypes characterized by increased telomere recombination and length are observed [90]. The capacity of Rbl1 and Rbl2 to regulate telomere function has been further explored using mouse embryonic fibroblasts deficient in Rbl1 and Rbl2, where markedly elongated telomeres were observed in the absence of increased telomerase activity and the retention of their end-capping function. Taken together, these data confirm the role of the Rb1 family in the regulation of telomere length in mammalian cells [91], suggesting that FTO may influence mammalian TL regulation processes.

## Conclusion

**Fig. 1:**
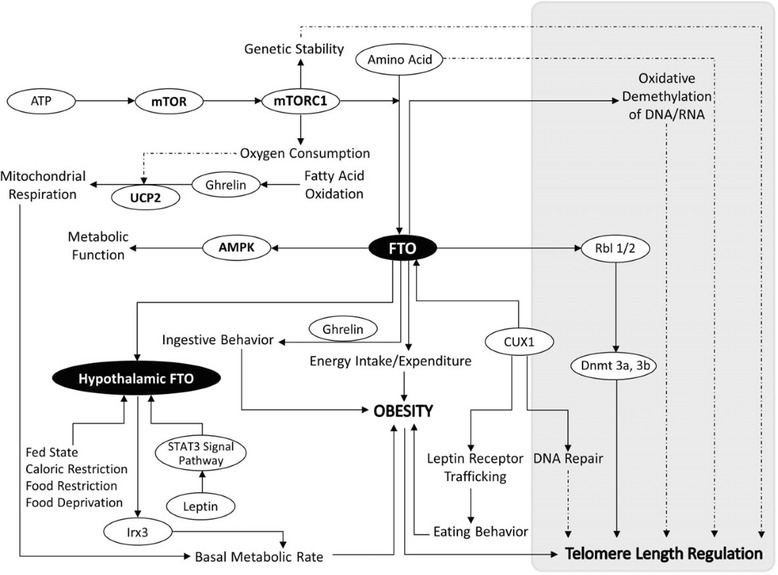
FTO gene interacts with telomere length and obesity. FTO interacts with uncoupling protein 2 (UCP2), AMP-activated protein kinase (AMPK), retinoblastoma-like 2 protein (RBL2), Iroquois homeobox protein 3 (IRX3), cut like homeobox 1 (CUX1) and mammalian target of rapamycin complex 1 (mTORC1). These interactions are important for dietary behavior regulation and cellular nutrient sensing. Additionally, the hypothesis is presented that the FTO genotype may influence telomere regulation. Bold arrow means there is published evidence; dotted arrow means there is rational speculation but without published evidence

We have previously reviewed the bi-directional interactions between diabetes and short telomeres [16]. We suggest in this review the possibility that FTO genotypes may be associated with genetic aging, i.e. shorter telomeres (Fig. 1). Dlouha et al. reported that carriers of at least one FTO risky (rs17817449 G) allele, is associated with shorter telomeres in middle age women [14]. Additionally, Zhou and colleagues found the relationship between FTO obesity-related risk allele (rs9939609 A) and shorter telomere length only in the high, but not low, FTO methylation levels in non-diabetics [11].

Importantly, we have discussed downstream genes and bi-directional feedback loops that influence obesity outcomes across FTO genotypes. FTO feedback loops may in part also be involved in telomere regulation. Certainly we have previously highlighted a role for UCP2 in telomere regulation [92] and currently in this review we relate UCP2 to obesity regulation. Bell et al. identified an FTO obesity susceptibility haplotype is associated with increases in methylation of the FTO gene [88]. Further well-designed and detailed studies in humans and animals are required to explore biochemical and functional roles of FTO genotypes and interactions with FTO epigenetic modification. We suspect these epigenetic FTO interactions with the FTO gene will have modifiable effects on obesity and telomere attrition.

## Abbreviations

2-OG: 2-oxoglutarate
AA: Amino acid
AKT/PKB: protein kinase B
AKTIP: AKT interacting protein
AMPK: AMP-Activated protein kinase
ATP: Adenosine triphosphate
CUX1: Cut like homeobox 1
DEPTOR: DEP-domain containing mTOR-interacting protein
FTO: Fat mass and obesity-associated (FTO) gene
GCN2: General control non-depressible 2
GWAS: Genome wide association study
Lepob: Leptin-knockout mice
LepRb: Leptin Receptor Long Isoform
Leprdb: Leptin receptor-mutated mice
mTOR: mammalian target of rapamycin
mTORC1: mammalian target of rapamycin complex 1
RPGRIP1L: Retinitis pigmentosa GTPase regulator interacting protein 1 like
TET: Ten-Eleven-Translocation
TL: Telomere length
TORC1: Target of rapamycin complex 1
UCP2: Uncoupling protein 2
